# Occupational Exposure to Asbestos and Ovarian Cancer: A Meta-analysis

**DOI:** 10.1289/ehp.1003283

**Published:** 2011-06-03

**Authors:** M. Constanza Camargo, Leslie T. Stayner, Kurt Straif, Margarita Reina, Umaima Al-Alem, Paul A. Demers, Philip J. Landrigan

**Affiliations:** 1 Division of Epidemiology and Biostatistics, University of Illinois, Chicago, Illinois, USA; 2 International Agency for Research on Cancer, Lyon, France; 3 Occupational Cancer Research Centre, Cancer Care Ontario, Toronto, Ontario, Canada; 4 Department of Preventive Medicine, Mount Sinai School of Medicine, New York, New York, USA

**Keywords:** asbestos, chrysotile, crocidolite, meta-analysis, ovarian cancer, SMR

## Abstract

Objective: A recent Monographs Working Group of the International Agency for Research on Cancer (IARC) concluded that there is sufficient evidence for a causal association between exposure to asbestos and ovarian cancer. We performed a meta-analysis to quantitatively evaluate this association.

Data sources: Searches of PubMed and unpublished data yielded a total of 18 cohort studies of women occupationally exposed to asbestos.

Data extraction: Two authors independently abstracted data; any disagreement was resolved by consulting a third reviewer.

Data synthesis: All but one study reported standardized mortality ratios (SMRs) comparing observed numbers of deaths with expected numbers for the general population; the exception was a study that reported standardized incidence ratios. For simplicity, we refer to all effect estimates as SMRs. The overall pooled SMR estimate for ovarian cancer was 1.77 (95% confidence interval, 1.37–2.28), with a moderate degree of heterogeneity among the studies (*I*^2^ = 35.3%, *p* = 0.061). Effect estimates were stronger for cohorts compensated for asbestosis, cohorts with estimated lung cancer SMRs > 2.0, and studies conducted in Europe compared with other geographic regions. Effect estimates were similar for studies with and without pathologic confirmation, and we found no evidence of publication bias (Egger’s test *p*-value = 0.162).

Conclusions: Our study supports the IARC conclusion that exposure to asbestos is associated with increased risk of ovarian cancer.

In 2008, cancer of the ovary represented the second leading cause of gynecologic cancer death worldwide (Ferlay et al. 2010). The geographical distribution of ovarian cancer is characterized by wide international variation. Highest rates are observed in North America and Northern Europe. In the United States, white women have higher incidence and mortality rates than do other racial and ethnic groups (Horner et al. 2009). Although the etiology of ovarian cancer is not well understood, multiparity, lactation, oral contraceptive use, and tubal ligation or hysterectomy are inversely associated with risk (Permuth-Wey and Sellers 2009; Sueblinvong and Carney 2009), whereas estrogen-only menopausal therapy, tobacco smoking, and other environmental, occupational, and genetic factors are positively associated with ovarian cancer (Antoniou et al. 2000; Grosse et al. 2009; Secretan et al. 2009; Shen et al. 1998).

Approximately 125 million people around the world work in environments in which they are exposed to asbestos, and at least 90,000 people die from asbestos-related lung cancer, mesothelioma, or asbestosis every year (Burki 2009). Asbestos exposure has been identified in some previous reviews as a possible risk factor for ovarian cancer (Hankinson and Danforth 2006; Ness and Cottreau 1999; Shoham 1994). However, this association has not been widely recognized. Perineal use of talc, which may in some formulations contain asbestiform or talc mineral fibers, has also been associated with ovarian cancer in a number of studies (Baan et al. 2006; Langseth et al. 2008).

The association between ovarian cancer risk and asbestos exposure was addressed by a Monographs Working Group that was convened in March 2009 by the International Agency for Research on Cancer (IARC). After considering the potential role of chance, confounding, and other forms of bias, the working group concluded that the evidence is sufficient for a causal association between occupational exposure to asbestos and ovarian cancer (Straif et al. 2009). To more fully evaluate and characterize this association, we performed a meta-analysis.

## Materials and Methods

We searched for studies of workers exposed to asbestos published in any language before March 2010 using PubMed software to search Medline (U.S. National Library of Medicine, Bethesda, MD). Combinations of the following keywords were used: “ovarian cancer,” “cancer of the ovary,” “asbestos,” “chrysotile,” “crocidolite,” “mortality,” “standardized mortality ratio” (SMR), “incidence,” “standardized incidence ratio” (SIR), “cancer,” “mesothelioma,” “cohort,” “female,” and “women.” In addition, we searched major cohorts of asbestos-exposed workers for data on ovarian cancer. References cited in the selected articles were also considered. Two investigators in our team independently reviewed the articles and extracted the data; any disagreement was resolved by consulting a third reviewer. We incorporated into the meta-analysis all studies of women who were occupationally exposed to asbestos meeting the following two criteria: *a*) an estimate of relative risk (i.e., SMRs or SIRs) for ovarian cancer or data allowing such estimates to be derived were presented, and *b*) the study was of a population with clear and unequivocal evidence of occupational exposure to asbestos such as asbestos cement and textile workers; asbestos miners and millers; friction material, insulator, and insulation board manufacturers; and workers compensated for asbestosis. Population- or hospital-based case–control studies that were based on jobs and industries with only limited documentation of asbestos exposures were excluded (Langseth and Kjaerheim 2004; Rosenblatt et al. 1992; Shu et al. 1989).

The following information was recorded for each study: first author, journal, geographic region of the cohort, year of publication, outcome (mortality or incidence), overall number of women, duration of follow-up, total person-years of observation, period of employment, industry sector, type of asbestos, SMR or SIR and 95% confidence interval (CI) for ovarian cancer (for simplicity, we refer to all effect estimates as SMRs), observed ovarian cancer cases or deaths, expected ovarian cancer cases or deaths, whether pathologic confirmation of the tumors was conducted, potential confounding variables adjusted for, total number of deaths, total number of cancer cases, total number of peritoneal mesothelioma cases, SMRs for lung cancer, and whether workers received compensation for asbestosis. In addition, data on national incidence rates for ovarian cancer were obtained from GLOBOCAN 2008 estimates for individual countries (Ferlay et al. 2010).

*Statistical analysis.* Based on the reported CIs, we estimated the standard errors (SEs) for the ln(SMR) or the ln(SIR) given by the formula SE = [ln(upper limit) – ln(lower limit)] ÷ (2 × *Z*_1–α/2_), where for a 95% CI, *Z*_1–α/2_ equals 1.96 (Bradburn 2004). For the studies for which the 95% CI was not reported, we calculated them by the Fischer’s exact method using the observed deaths and expected deaths reported in the articles (Dean et al. 2010).

Overall pooled SMR estimates and their corresponding 95% CIs were obtained using fixed-effects (Mantel–Haenszel method) and random-effects (DerSimonian and Laird method) methods (Harris 2008). Given the significant amount of heterogeneity, only the random-effects estimates are presented. Meta-regression techniques were used to examine the extent to which one or more of the following variables might explain heterogeneity: outcome (mortality or incidence), cohort size (< 500, 500–1,000, or > 1,000 women), follow-up period (< 25 or ≥ 25 years), geographic region (Europe vs. United States and Australia), national ovarian cancer incidence rate (< 12 and ≥ 12 cases/100,000 women), type of industry (mining, textiles, cement, gas mask manufacturing, mixed, or other), type of asbestos (chrysotile, crocidolite, or mixed), compensation for asbestosis (yes or no), magnitude of the SMR for lung cancer (≤ 2.0 or > 2.0), and pathological confirmation (yes or no). Between-study heterogeneity was assessed using the *Q* and *I*^2^ statistics, with *P_Q_* < 0.10 or *I*^2^ > 25% indicating significant heterogeneity (Higgins and Thompson 2002; Higgins et al. 2003). A log-likelihood test was used to measure the improvement in fit when explanatory variables were included compared with the null model. The proportion of between-study variance explained by a specific variable was described using adjusted *R*^2^ estimates that could be negative for variables that explain less of the heterogeneity than would be expected by chance (Sterne 2009). Publication bias was investigated by visual inspection of Begg’s funnel plots and formally tested using Egger’s regression asymmetry method. The influence of individual studies was assessed by sequentially dropping each one before pooling study-specific estimates. A visual impression of the amount of heterogeneity was explored using Galbraith plots to identify the studies contributing to heterogeneity (the *z*-statistic Φ/SE_Φ_ was plotted against the reciprocal SE 1/SE_Φ_, where Φ was the effect estimated from the individual study and SE_Φ_ was its SE).

Tumor misclassification could lead to both false-positive and false-negative diagnoses. Nielsen et al. (1994), based on a large series of peritoneal mesotheliomas, estimated that 16% of cases were misdiagnosed as ovarian cancer cases. Assuming that a similar proportion of peritoneal mesotheliomas are misclassified as ovarian cancer cases, we evaluated the effect of misclassification on the overall pooled SMR estimate by removing 20% of ovarian cancer cases from every study and repeating the meta-analysis.

As a proxy for a dose–response analysis, we performed a meta-analysis that combined the results of women with the highest occupational asbestos exposure from studies reporting either duration or cumulative exposure.

The meta-analyses were performed with Stata software (version 10; StataCorp LP, College Station, TX, USA) using a combination of available macros (Sterne 2009). Meta-regression analyses were performed using the Proc Mixed in SAS (version 9.1; SAS Institute Inc., Cary, NC, USA). A *p*-value < 0.05 was considered statistically significant for all tests except for the heterogeneity.

## Results

**Figure 1:**
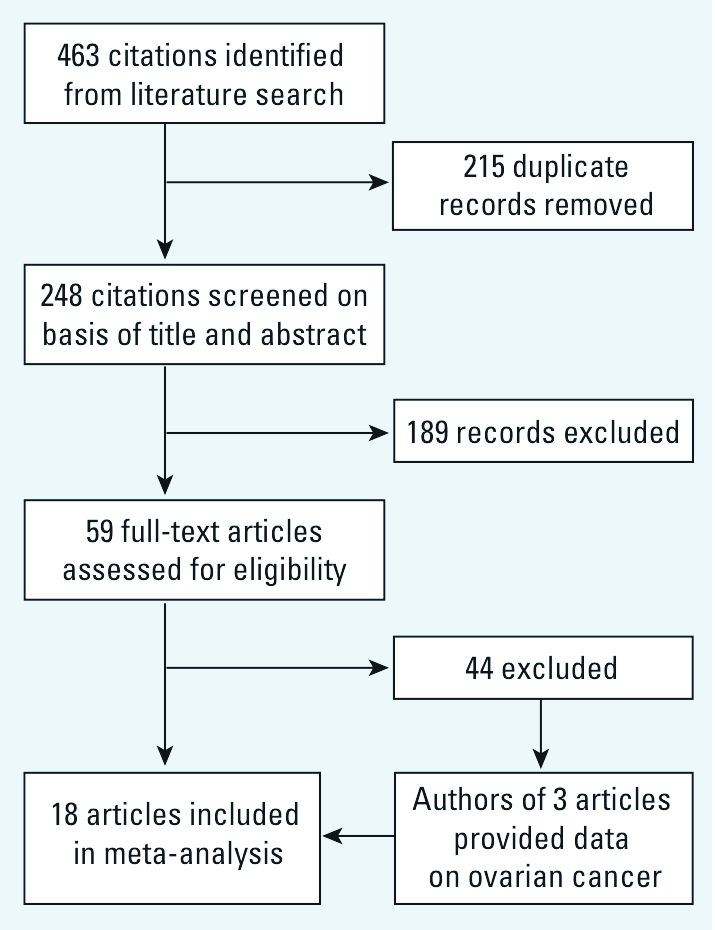
Flow chart of the meta-analysis.

*Literature search.* We identified 15 references that met the criteria for inclusion in the meta-analysis (Acheson et al. 1982; Berry et al. 2000; Gardner et al. 1986; Germani et al. 1999; Harding et al. 2009; Magnani et al. 2008; Mamo 2004; McDonald et al. 2006; Newhouse and Sullivan 1989; Pira et al. 2007; Reid et al. 2009; Rösler et al. 1994; Szeszenia-Dabrowska et al. 2002; Tarchi et al. 1994; Wilczyńska et al. 2005) (Figure 1). In addition, we were able to obtain results from the investigators of three cohort studies that had not previously reported findings for ovarian cancer (Clin et al. 2009; Hein et al. 2007; Loomis et al. 2009). Two of the identified 15 articles (Acheson et al. 1982; Germani et al. 1999) reported findings from two distinct cohorts, and thus we analyzed data on 20 distinct populations. The data were from the most recently published reference for each cohort, except when results for high- exposure groups were not reported in the latest publication (Pira et al. 2005; Wignall and Fox 1982).

We excluded studies conducted among workers who were predominantly exposed to other known or suspected carcinogens but also had some potential for exposure to asbestos (Atkinson et al. 2004; Beall et al. 2005; Boice et al. 1999; Bulbulyan et al. 1999; Coggon et al. 1997; Costantini et al. 1994; Langseth and Andersen 1999; Lewis et al. 2003; Richardson et al. 2007; Settimi et al. 1999; Vasama-Neuvonen et al. 1999; Ward et al. 1994).

**Figure 2:**
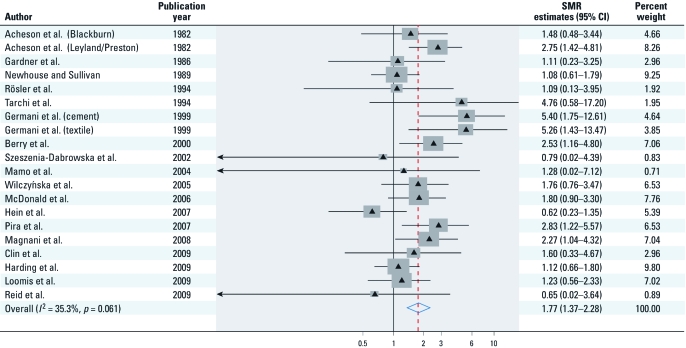
SMR estimates and 95% CIs of ovarian cancer associated with occupational exposure to asbestos. Weights are from random-effects analysis. Study-specific SMRs are shown as triangles, with the size of the boxes being inversely proportional to the study-specific SMR variance. Horizontal lines represent 95% CIs for the study-specific SMRs. The pooled SMR is shown as a diamond. The middle of the diamond corresponds to the SMR, and the width of the diamond represents the 95% CI. The vertical dashed red line provides a visual comparison of the pooled SMR with the corresponding study-specific SMRs.

Table 1 summarizes the main characteristics of the selected studies. Only one study reported findings for ovarian cancer incidence (Reid et al. 2009); the remaining studies were based on mortality. We included a total of 125 ovarian cancer deaths and one incident cancer case in our main analysis. SMR estimates reported by the individual studies ranged from 0.62 to 5.40 (Figure 2). Most of the studies had been carried out in Europe (*n* = 15). Two studies were conducted in the United States, and one in Australia. Although some cohorts included only females, the majority included both males and females (*n* = 14). The industries involved included the manufacture of textiles, mining, cement production, manufacture of friction material, and manufacture of gas masks. Some industries included manufacturing of a wide range of goods containing asbestos. Only two studies conducted pathologic review of cases (Magnani et al. 2008; Reid et al. 2009). Peritoneal mesothelioma cases were reported in five of the six studies that reported a significant excess mortality from ovarian cancer (Acheson et al. 1982; Berry et al. 2000; Germani et al. 1999; Magnani et al. 2008; Pira et al. 2007), and we did not include these when deriving SMRs for ovarian cancer.

**Table 1:** Study characteristics.

National incidence rate for ovarian cancer*a*	Ovarian cancer results	
	Reference	Country	Outcome studied	Industry type	Asbestos type	Cohort size	Period of employment	Follow-up period	Person-years	Total deaths	Total cancers	Lung cancer SMR	Observed/expected deaths or cases	SMR or SIR (95% CI)
Acheson et al. 1982	United Kingdom	12.8	Mortality	Gas mask assemblers (in Leyland and Preston)	Crocidolite	757	1927–1939	1951–1980	18,781	219	66	2.41*b*	12/4.4	2.75 (1.42–4.81)
			Mortality	Gas mask assemblers (in Blackburn)	Chrysotile	570	1927–1945	1951–1980	14,324	177	44	1.45*b*	5/3.4	1.48 (0.48–3.44)
Gardner et al. 1986	United Kingdom	12.8	Mortality	Cement	Chrysotile	657	1941–1954	1941–1984	—	102	26	1.42	3/2.7	1.11 (0.23–3.25)
Newhouse and Sullivan 1989	United Kingdom	12.8	Mortality	Production of friction materials	Chrysotile	4,345	1941–1979	1941–1986	—	522	148	0.66*b*	11/10.1	1.08 (0.61–1.79)
Rösler et al. 1994	Germany	10.0	Mortality	Mixed (mainly textile)	Mixed (mainly chrysotile)	616	—	1977–1988	6,236	64	32	3.39	2/1.8	1.09 (0.13–3.95)
Tarchi et al. 1994	Italy	8.7	Mortality	Mining	Chrysotile	120	—	1965–1989	—	28	8	4.14*c*	2/0.42	4.76 (0.58–17.2)
Germani et al. 1999	Italy	8.7	Mortality	Textile (compensated for asbestosis)	Chrysotile	276	—	1980–1997	3,761	123	40	6.82	4/0.76	5.26 (1.43–13.47)
				Cement (compensated for asbestosis)	Mixed (mainly crocidolite)	278	—	1980–1997	3,932	129	54	2.39	5/0.93	5.40 (1.75–12.61)
Berry et al. 2000	United Kingdom	12.8	Mortality	Textile and prefabricated cement pipes	Mixed	700	1936–1942	Up to June, 1980	17,146	—	129	7.46	9/3.56	2.53 (1.16–4.80)
Szeszenia-Dabrowska et al. 2002	Poland	12.6	Mortality	Mixed (compensated for asbestosis, mainly asbestos processing plants)	Mixed	490	1970–1997 (diagnosis period)	Up to Dec. 31, 1999	—	121	34	6.21	1/1.27	0.79 (0.02–4.39)
Mamo 2004	Italy	8.7	Mortality	Textile	Chrysotile	645	1951–1978	1981–1995	7,450	84	36	5.23	1/0.78	1.28 (0.02–7.12)
Wilczyn´ska et al. 2005	Poland	12.6	Mortality	Mixed	Mixed	1,201	1945–1980	Up to Dec. 31, 1999	—	414	124	2.09	8/4.5	1.76 (0.76–3.47)
McDonald et al. 2006	United Kingdom	12.8	Mortality	Gas mask assemblers	Crocidolite	1,073	1940–1944	1963–2003	—	—	—	2.73*d*	10/5.6	1.80 (0.9–3.3)
Hein et al. 2007	United States	8.8	Mortality	Textile	Chrysotile	1,265	1940–1965	1979–2001	49,922	709	169	2.22*c*	6/9.68	0.62 (0.23–1.35)
Pira et al. 2007	Italy	8.7	Mortality	Textile	Mixed	1,077	1946–1984	Up to Dec. 31, 2004	36,886	254	130	6.5	8/2.8	2.83 (1.22–5.57)
Magnani et al. 2008	Italy	8.7	Mortality	Cement	Mixed	777	1912–1986	1965–2003	22,367	371	169	2.21	9/4	2.27 (1.04–4.32)
Loomis et al. 2009	United States	8.8	Mortality	Textile	Chrysotile	1,795	1950–1973	Up to Dec. 31, 2003	59,949	608	160	1.73*c*	9/7.34	1.23 (0.56–2.33)
Reid et al. 2009	Australia	7.7	Incidence	Mining and milling	Crocidolite	416	1943–1966	1960–2006	—	—	—	—	1/1.54	0.65 (0.02–3.64)
Harding et al. 2009	United Kingdom	12.8	Mortality	Mixed	Mixed	4,495	—	1971–2005	103,394	—	—	—	17/15.2	1.12 (0.66–1.80)
Clin et al. 2009	France	7.7	Mortality	Mixed	Mixed	420	—	1978–2004	—	—	11	—	3/1.88	1.60 (0.33–4.67)
––, Not available. **a**Cases per 100,000 women, GLOBOCAN 2008 (Ferlay et al. 2010). **b**Lung and pleura are included. **c**Trachea, bronchus, and lung are included. **d**From Wignall and Fox (1982).														

*Pooled SMR estimate.* Figure 2 shows the SMR estimates and 95% CIs from the individual studies and the pooled SMR estimate based on a random-effects model. The average pooled estimate for ovarian cancer among asbestos exposed women was 1.77 (95% CI, 1.37–2.28), with a moderate degree of heterogeneity among the studies (*I*^2^ = 35.3%, *p* = 0.061).

*Exploring between-study heterogeneity.* The evidence for heterogeneity was strong enough to warrant investigation of potential explanatory factors. Table 2 presents the findings from the meta-regression models for all covariates. Compensation for asbestosis, magnitude of the SMR for lung cancer, geographic region, and sample size were each statistically significant predictors of the pooled ovarian cancer SMR based on the –2 log-likelihood test. The simultaneous inclusion of these predictors in a regression model virtually eliminated any degree of heterogeneity (*I*^2^ = 0%, *R*^2^ = 100%). Pooled SMR estimates were increased for cohorts that were compensated for asbestosis (SMR = 4.67; 95% CI, 2.28–9.54) compared with cohorts that were not compensated (SMR = 1.60; 95% CI, 1.28–2.00); cohorts that reported an SMR for lung cancer > 2.0 (SMR = 2.25; 95% CI, 1.64–3.07) compared with other cohorts (SMR = 1.18; 95% CI, 0.81–1.72); European cohorts (SMR = 1.95; 95% CI, 1.51–2.51; 15 studies representing 17 cohorts) compared with cohorts from the United States and Australia, for which there appeared to be no increase in ovarian cancer mortality (SMR = 0.92; 95% CI, 0.54–1.59; three studies); and for smaller cohorts compared with larger cohorts. We found suggestive albeit nonsignificant evidence for variation by type of asbestos. Pooled SMRs were larger for cohorts exposed predominantly to crocidolite (SMR = 2.18; 95% CI, 1.40–3.37) or mixed asbestos (SMR = 2.00; 95% CI, 1.41–2.84) than for cohorts exposed to chrysotile (SMR = 1.40; 95% CI, 0.88–2.21). Geographic region was no longer a significant predictor of the pooled SMR when we excluded six European studies of gas mask assemblers (*n* = 3) and cohorts compensated for asbestosis (*n* = 3; data not shown).

**Table 2:** Pooled random-effects model-based SMR estimates and 95% CIs of ovarian cancer associated with asbestos exposure by study characteristics.

Covariable	*na*	Pooled SMR (95% CI)	*I*^2 ^(%)	*PQ*	*P*_LLR_	Adjusted *R*^2 ^(%)*b*
No covariables		20		1.77 (1.37–2.28)		35.3		0.06		—		
Type of outcome												
Incidence		1		—		—		—		0.48		–1.8
Mortality		19		1.79 (1.38–2.31)		37.6		0.05				
Type of asbestos												
Chrysotile		8		1.40 (0.88–2.21)		39.2		0.12		0.26		17.8
Crocidolite		3		2.18 (1.40–3.37)		0.0		0.42				
Mixed		9		2.00 (1.41–2.84)		29.9		0.18				
Compensation for asbestosis												
Yes		3		4.67 (2.28–9.54)		0.0		0.41		0.01		52.0
No		17		1.60 (1.28–2.00)		17.6		0.25				
Geographic region												
Europe		17		1.95 (1.51–2.51)		28.2		0.13		0.03		26.2
United States and Australia		3		0.92 (0.54–1.59)		0.0		0.48				
Pathology confirmation												
Yes		2		2.08 (1.05–4.14)		0.0		0.36		1.0		–14.0
No		18		1.76 (1.34–2.31)		39.7		0.04				
Follow-up period in years												
< 25		6		1.83 (0.81–4.16)		67.2		0.01		1.0		–15.2
≥ 25		14		1.73 (1.38–2.16)		7.9		0.37				
Sample size												
< 500		6		3.37 (1.82–6.25)		9.0		0.36		0.01		100.0
500–1,000		7		2.16 (1.54–3.03)		0.0		0.80				
> 1,000		7		1.35 (0.99–1.84)		34.0		0.17				
SMR for lung cancer												
≤ 2.0		4		1.18 (0.81–1.72)		0.0		0.96		< 0.001		89.7
> 2.0		13		2.25 (1.64–3.07)		30.5		0.14				
No data		3		1.15 (0.73–1.82)		0.0		0.81				
Type of industry												
Mining		2		2.27 (0.34–14.97)		36.7		0.21		0.55		–20.0
Textile		5		1.73 (0.81–3.70)		65.0		0.02				
Cement		3		2.56 (1.17–5.58)		47.8		0.15				
Gas mask manufacturing		3		2.10 (1.40–3.15)		0.0		0.48				
Mixed		6		1.50 (1.07–2.10)		0.0		0.56				
Others		1		—		—		—				
Ovarian cancer incidence rate*c*												
< 12		11		2.02 (1.27–3.21)		46.2		0.05		0.58		–5.9
≥ 12		9		1.59 (1.22–2.06)		14.5		0.31				
Abbreviations: ––, not applicable; *PQ*, *p*-value for the heterogeneity test; *P*_LLR_, *p*-value of the log-likelihood ratio test (when compared with the model containing no covariables). SMR includes data from one cohort that reported an SIR. **a**Number of cohorts included. **b**Estimates can be negative if the covariable explains less of the heterogeneity than would be expected by chance (Sterne 2009). **c**National rate as cases per 100,000 women, GLOBOCAN 2008 (Ferlay et al. 2010).												

*Influence of individual studies.* The pooled SMR estimates were relatively robust to the exclusion of any one study from the overall meta-analysis and did not change by > 10% (data not shown).

Based on a visual impression of the amount of heterogeneity across all studies (data not shown), a study by Germani et al. (1999), conducted in cement workers, and a study by Hein et al. (2007) were the greatest contributors to between-study variation. SMRs for both studies were outside the 95% CI of the regression line, in opposite directions. The pooled SMR estimates for ovarian cancer excluding the cement worker study by Germani et al. (1999), the Hein et al. (2007) study, or both were 1.67 (95% CI, 1.32–2.11), 1.86 (95% CI, 1.47–2.36), and 1.74 (95% CI, 1.41–2.16), respectively. Heterogeneity was reduced when we removed both of these studies (*I*^2^ = 11%, *p* = 0.323).

**Figure 3:**
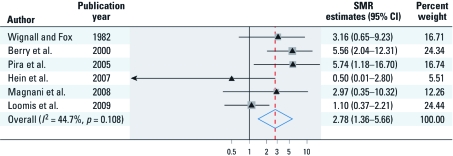
SMR estimates and 95% CIs of ovarian cancer associated with high occupational exposure to asbestos. Weights were from random-effects analysis. Study-specific SMRs are shown as triangles, with the size of the boxes being inversely proportional to the study-specific SMR variance. Horizontal lines represent 95% CIs for the study-specific SMRs. The pooled SMR is shown as a diamond. The middle of the diamond corresponds to the SMR, and the width of the diamond represents the 95% CI. The vertical dashed red line provides a visual comparison of the pooled SMR with the corresponding study-specific SMRs.

*Analysis of highly exposed groups.* Estimates of cumulative or duration of exposure among asbestos-exposed workers were reported for only six studies (Berry et al. 2000; Hein et al. 2007; Loomis et al. 2009; Magnani et al. 2008; Pira et al. 2005; Wignall and Fox 1982; Table 3). The pooled SMR estimate of ovarian cancer based on these six high-exposure groups was 2.78 (95% CI, 1.36–5.66; Figure 3). We found a moderate degree of heterogeneity across all studies (*I*^2^ = 45%, *p* = 0.108).

**Table 3:** SMR estimates of studies included in the analysis of highly exposed groups.

Ovarian cancer results		
Reference	Country	Industry type	Observed/expected deaths	SMR (95% CI)	Variable (highest category)
Pira et al. 2005, 2007		Italy		Textile		3/0.53		5.74 (1.18–16.7)		Duration of employment (≥ 10 years)
Berry et al. 2000		United Kingdom		Textile and prefabricated cement pipe		5/0.9		5.56 (2.04–12.31)		Exposure and duration (severe exposure with > 2 years of duration)
Wignall and Fox 1982 McDonald et al. 2006		United Kingdom		Gas mask assemblers		3/0.95		3.16 (0.65–9.23)		Duration of employment (≥ 1 year)
Loomis et al. 2009		United States		Textile		6*a*/5.45		1.10 (0.37–2.21)		Cumulative exposure (≥ 120 fiber-days/ml)
Hein et al. 2007		United States		Textile		1/1.99		0.50 (0.01–2.80)		Cumulative exposure and duration (> 30 years of employment and ≥ 5,479 fiber-days/mL)
Magnani et al. 2008		Italy		Cement		2/0.7		2.97 (0.35–10.32)		Duration of exposure (≥ 30 years)
**a**Three ovarian cancer cases from the fourth plant were omitted.										

*Influence of tumor misclassification.* Results of the sensitivity analysis assuming that 20% of the cases were misclassified as ovarian cancers suggested some attenuation of the pooled effect estimate (SMR = 1.42; 95% CI, 1.11–1.82), with a moderate degree of heterogeneity among the studies (*p* = 0.104; *I*^2^ = 29.7%).

**Figure 4:**
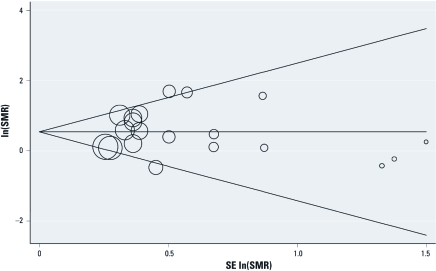
Begg’s funnel plot with pseudo-95% CIs for ovarian cancer SMRs associated with occupational exposure [natural log (In)] of the pooled SMR of the 20 cohorts = 0.57].

*Assessing publication bias.* Figure 4 presents the Begg’s funnel plot including all 20 populations. Egger’s test produced a *p*-value of 0.162, which provides little evidence of publication bias.

## Discussion

The association between asbestos and ovarian cancer has been assessed here among studies of workers in which a major portion of the cohort is presumed to have been exposed to asbestos. Our results demonstrate an increase in the pooled estimate (SMR = 1.77; 95% CI, 1.37–2.28) for ovarian cancer in relation to exposure to asbestos.

The magnitude of the pooled estimate is similar to that reported by Edelman (1992), who included six studies conducted in the United Kingdom published before 1989 (pooled SMR = 1.65; 95% CI, 1.27–2.16). They concluded, however, that despite the positive and significant association, there was insufficient information to infer that ovarian cancers were caused by occupational exposure to asbestos because of concerns about tumor misclassification, inappropriate comparison populations, and the failure to take into account for known risk factors. A more recent meta-analysis by Li et al. (2004) of three studies published before February 2003 of workers exposed only to chrysotile found a nonsignificant association (pooled SMR = 1.81; 95% CI, 0.61–5.36; *P_Q_* < 0.05). These previous meta-analyses by Edelman (1992) and Li et al. (2004) did not evaluate sources of between-study variability. Our analysis addressed heterogeneity and was based on studies included in the published meta-analyses, other available data, and more recent publications.

Our search for sources of heterogeneity revealed that SMRs based on European cohorts suggested stronger effects of occupational asbestos exposure than did estimates based on cohorts from other geographic regions. This geographic variation seems to have been driven by data from studies of Italian and Polish women compensated for asbestosis and of United Kingdom women who manufactured gas masks, who most likely had been exposed to high levels of asbestos fibers. Indeed, the estimated effect of geographic region was no longer significant when we excluded both subsets of studies. Our analysis of heterogeneity also suggested that stratification according to sample size reduced heterogeneity. The observation that the smaller the cohort size, the larger the SMR was related to limited cohort size (< 500) in the three studies of women compensated for asbestosis. Sample size was no longer an important predictor once we dropped the studies of women with asbestosis and gas mask production.

The results from the analysis of highly exposed workers indicate a stronger effect than among all workers combined. Similarly, occupational exposure was more strongly associated with ovarian cancer among cohorts with a lung cancer SMR > 2.0.

We found a suggestive but nonsignificant association between asbestos type and the pooled ovarian cancer SMR. Cohorts predominantly exposed to crocidolite or mixed asbestos showed larger SMRs than did those exposed only to chrysotile asbestos. This finding is similar to what Stayner et al. (1996) found for mesothelioma. In addition, the nonsignificant SMR based on the eight cohorts with exposure to chrysotile asbestos only seems to confirm the results by Li et al. (2004) based on three studies.

The observed overall heterogeneity among studies seemed to be explained by two cohorts (Germani et al. 1999; Hein et al. 2007). The study by Germani et al. (1999) of 278 Italian cement industry workers compensated for asbestosis (with mixed exposure, mainly crocidolite) reported a very large increase in mortality for ovarian cancer (SMR = 5.4; 95% CI, 1.75–12.61), possibly because the study was limited to subjects with asbestosis who were likely highly exposed. The study by Hein et al. (2007) of 1,265 U.S. women exposed to chrysotile in a textile plant reported a nonsignificant decrease in mortality for ovarian cancer (SMR = 0.62; 95% CI, 0.23–1.35).

Pathophysiologic mechanisms by which asbestos may confer susceptibility to ovarian cancer have been proposed. They relate mainly to the hypothesis that the persistent presence of asbestos fibers in ovarian tissue causes chronic inflammation. This hypothesis is supported by reports of asbestos fibers in the ovaries of women occupationally and non-occupationally exposed to asbestos (Heller et al. 1996; Langseth et al. 2007). The mechanism of transportation of asbestos fibers to the ovary is not clearly understood. Retrograde movement of particles through the reproductive tract to the ovaries has been suggested (Baan et al. 2006; Heller et al. 1996). Alternatively, blood-borne or lymph-borne fibers could penetrate to the ovary through the mesothelium. This mechanism is supported by the findings of *in vivo* studies in animal models demonstrating changes in the ovaries of guinea pigs and rabbits after peritoneal injection of asbestos fibers (Graham and Graham 1967). In addition, perineal exposure to talc, which may in the past have contained asbestos or talc fibers, has also been associated in a number of studies with an increased risk of ovarian cancer (Baan et al. 2006; Langseth et al. 2008).

A major concern in interpreting our findings is that until recently it has been very difficult to distinguish pathologically between peritoneal mesothelioma and ovarian cancer (Kannerstein et al. 1977). In fact, misdiagnosis of cases of peritoneal mesotheliomas as ovarian cancer was previously identified in two studies that included pathologic review (Newhouse et al. 1972; Wignall and Fox 1982). Reid et al. (2009) examined the potential for misclassification by reviewing pathologic material on ovarian, colon, and peritoneal cancer and reported that none of the cancer specimens had been misclassified in their study. We did not observe a difference in pooled SMRs between studies with and without pathologic confirmation, but the power of this test was limited because there were only two studies with pathologic confirmation (Magnani et al. 2008; Reid et al. 2009). We also did not observe a large attenuation of the association when we assumed that 20% of the ovarian cancer cases in each study were misclassified. Given our findings from this sensitivity analysis, it would seem unlikely that the association between occupational asbestos exposure and ovarian cancer could be fully explained by tumor misdiagnosis.

Our meta-analysis mainly represents studies conducted in developed areas, particularly among European populations. It is possible that studies conducted in other geographic regions (e.g., developing countries) may be available through other biomedical literature databases. However, the previous meta-analysis by Li et al. (2004), which searched Chinese literature, found no articles on ovarian cancer published in that language.

A further limitation of our analysis was its inability to account for nonoccupational risk factors for ovarian cancer other than age. Differences in the definitions of duration or latency of asbestos exposure measures prevented a proper evaluation of a dose–response relationship. Although imperfect, our meta-analysis restricted to highly exposed women is compatible with an underlying dose–response effect.

Finally, of even greater potential concern was the fact that some of the published studies failed to include findings for ovarian cancer or only reported results for cancers of the female genital organs. We identified 20 cohort studies of asbestos-exposed women that failed to report findings for ovarian cancer (Armstrong et al. 1988; Cheng and Kong 1992; Clin et al. 2009; Finkelstein 1989; Hein et al. 2007; Karjalainen et al. 1999; Knox et al. 1968; Loomis et al. 2009; Luberto et al. 2004; McDonald et al. 1980; Morinaga et al. 1991; Pang et al. 1997; Peto et al. 1977; Raffaelli et al. 2007; Raffn et al. 1989; Sichletidis et al. 2009; Smailyte et al. 2004; Sun et al. 2003; Woitowitz et al. 1986; Zhu and Wang 1993). Because of our familiarity with the authors, we were able to obtain unpublished results from three of these studies (Clin et al. 2009; Hein et al. 2007; Loomis et al. 2009). The remaining studies had, in general, a relatively small number of women or included short follow-up periods.

## Conclusion

The findings from this analysis are consistent with the hypothesis that exposure to asbestos is associated with an increased risk of ovarian cancer. Based on our sensitivity analysis, it appears unlikely that our results can be fully explained by misclassification of ovarian cancer and peritoneal mesothelioma or other sources of bias and confounding. Our results therefore support the conclusion by IARC that exposure to asbestos is causally associated with an increased risk of ovarian cancer.
